# Neutrophil-Derived CCL3 Is Essential for the Rapid Recruitment of Dendritic Cells to the Site of *Leishmania major* Inoculation in Resistant Mice

**DOI:** 10.1371/journal.ppat.1000755

**Published:** 2010-02-05

**Authors:** Mélanie Charmoy, Saskia Brunner-Agten, David Aebischer, Floriane Auderset, Pascal Launois, Geneviève Milon, Amanda E. I. Proudfoot, Fabienne Tacchini-Cottier

**Affiliations:** 1 Department of Biochemistry, WHO Immunology Research and Training Center, University of Lausannne, Epalinges, Switzerland; 2 Institut Pasteur, Département de Parasitologie et Mycologie, Unité d'Immunophysiologie et Parasitisme Intracellulaire, Paris, France; 3 Merck-Serono Geneva Research Center, Geneva, Switzerland; Imperial College London, United Kingdom

## Abstract

Neutrophils are rapidly and massively recruited to sites of microbial infection, where they can influence the recruitment of dendritic cells. Here, we have analyzed the role of neutrophil released chemokines in the early recruitment of dendritic cells (DCs) in an experimental model of *Leishmania major* infection. We show in vitro, as well as during infection, that the parasite induced the expression of CCL3 selectively in neutrophils from *L. major* resistant mice. Neutrophil-secreted CCL3 was critical in chemotaxis of immature DCs, an effect lost upon CCL3 neutralisation. Depletion of neutrophils prior to infection, as well as pharmacological or genetic inhibition of CCL3, resulted in a significant decrease in DC recruitment at the site of parasite inoculation. Decreased DC recruitment in CCL3^−/−^ mice was corrected by the transfer of wild type neutrophils at the time of infection. The early release of CCL3 by neutrophils was further shown to have a transient impact on the development of a protective immune response. Altogether, we identified a novel role for neutrophil-secreted CCL3 in the first wave of DC recruitment to the site of infection with *L. major*, suggesting that the selective release of neutrophil-secreted chemokines may regulate the development of immune response to pathogens.

## Author Summary

When infectious agents enter our body, neutrophils are the first cells recruited to the scene. In addition to their capacity to kill microbes, neutrophils secrete molecules that attract other cells also involved in immune defense, such as dendritic cells (DCs). Here, we investigate the secretion of DC-attracting chemokines by neutrophils after inoculation of mice with *Leishmania major*, a protozoan parasite that can cause long-lasting, skin ulcers in man. Following parasite inoculation, most inbred strains of mice (e.g.C57BL/6) develop self-resolving lesions, while in a few strains (e.g. BALB/c) lesions fail to heal. We report here that in “healer” C57BL/6 mice, higher numbers of DCs were attracted at the site of infection than in “non-healer” BALB/c mice. DC recruitment correlated with secretion by neutrophils of the chemokine CCL3, as indeed a markedly decreased DC recruitment was observed in C57BL/6 mice depleted of neutrophils or deprived of the capacity to produce CCL3. DC recruitment was restored upon transfer of normal neutrophils to CCL3 deficient mice. Our results reveal a crucial role for neutrophil-secreted CCL3 in early recruitment of DCs in *L. major*-infected “healer” mice, and suggest that the type of chemokine secreted by neutrophils will have consequences in the launching of pathogen-specific immune response.

## Introduction

Neutrophils rapidly accumulate at the site of microbial infection and recent evidence show that they play a major role in immunity to several pathogens. Neutrophils, through the early release of cytokines and chemokines, create a microenvironment critical for the shaping of the development of an antigen-specific immune response (reviewed in [1],[2]).

Analyzing the early mechanisms controlling dendritic cell migration to the skin will contribute to the understanding of the development of immunity against infections. To this end, the experimental murine model of infection with the protozoan parasite *Leishmania major* (*L. major*) was used. After parasite inoculation, most mouse strains, including C57BL/6 mice, are resistant to infection and develop a protective CD4^+^ Th1 immune response, while a few strains such as BALB/c mice are susceptible to infection and develop a CD4^+^ Th2 type of immune response (reviewed in [3]). Following infection with *L. major*, neutrophils are massively and equally recruited to the site of parasite inoculation in mice from both strains of mice [4],[5], and recently, early recruitment of neutrophils and their essential role in the development of *L. major* protective immune response was confirmed using mice infected in the ear through the bite of female sandflies [6]. Depletion of neutrophils prior to *Leishmania* inoculation was shown to modify the development of the CD4^+^ T helper immune response [5],[7],[8], however, the exact mechanism(s) involved in this early process remain(s) to be determined.

Once exposed to *L. major* promastigotes, neutrophils from mice resistant or susceptible to infection were reported to develop distinct phenotypes including differential expression of Toll-like receptors and cytokine secretion [9]. Neutrophils could therefore create a microenvironment in the skin and influence that of skin draining lymph node, determining the development of the antigen-specific immune response. Dendritic cells, the most efficient antigen presenting cells, will be critically involved in this process. Indeed, following infection with *L. major*, dendritic cells have been reported to be crucial in the resistance to infection [10],[11], reviewed in [12]. Thus, considering the massive presence of neutrophils recruited to the site of parasite inoculation within the first day of infection, we hypothesized that crosstalk between neutrophils and dendritic cells at the site of infection might shape the development of the *L. major* specific immune response. DCs present in the *L. major* inoculated skin are trafficking from the epidermis/dermis, or recruited from the blood or/and from the bone marrow. They may include Langerhans cells [13], dermal DCs [14], as well as the rapidly differentiating monocyte-derived DCs [15],[16].

In the present study, we have analyzed the role of neutrophil-derived chemokines in the recruitment/trafficking of dendritic cells in the skin, during the first days of infection with *Leishmania major*. Although several chemokines have been reported to attract immature DC, the particular role of neutrophil-secreted chemokines in this early process has been scantily investigated. We analyzed the secretion of CCL3, CCL4, CCL5 and CCL20, as these chemokines have been reported to be both secreted by neutrophils, and to recruit immature DCs [17],[18],[19],[20].

Our results indicate first, that neutrophils from C57BL/6 *L. major* resistant mice secrete significantly more CCL3 than BALB/c susceptible mice in response to *L. major* in vitro, and second, that CCL3 is the key chemokine involved in chemoattraction of immature DCs. Infected C57BL/6 mice displayed high levels of CCL3 one day post *L. major* inoculation in the ear dermis, and markedly more Langherans cells, dermal DCs and monocyte-derived DCs were recruited to the site of parasite inoculation than in infected BALB/c mice, an effect mediated by neutrophil-derived CCL3. The early neutralization of CCL3 or its absence in CCL3^−/−^ mice resulted in a delay in development of IFNγ secreting-Th1 cells, correlating with transient higher parasite load and tissue damage, a phenotype more sustained and statistically significant in CCL3^−/−^ mice. This identifies the CCL3 secreted by neutrophils during the first days of infection as a critical chemokine involved in the recruitment/trafficking of dendritic cells, which influences the subsequent development of the immune response.

## Results

### In presence of *L. major*, neutrophils produce CCL3 which chemoattracts immature bone marrow-derived DCs

**Figure 1:**
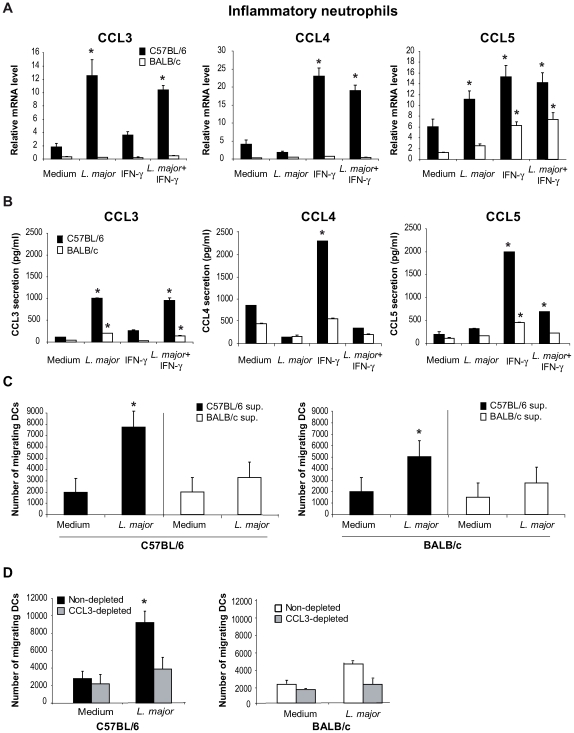
In presence of *L. major*, neutrophils produce CCL3 which chemo-attracts bone marrow-derived DCs *in vitro*. *L. major*–i.p. recruited C57BL/6 and BALB/c neutrophils were purified by MACS and incubated with medium, *L. major* (5∶1 parasite∶neutrophil ratio), IFNγ, or both. (A) Sixteen hours later, neutrophil CCL3, CCL4 and CCL5 mRNA levels were assessed by real-time quantitative RT-PCR, and (B) 24 hours after initiation of culture, chemokine content was measured in cell-free culture supernatants by ELISA. Data are the mean triplicate measurement ± SEM of neutrophil mRNA or chemokine content in the supernatants. *: p<0.05 compared to neutrophils cultured with medium only. (C) Supernatants from inflammatory neutrophils cultured in presence or in absence of *L. major* were tested for their chemotactic activity towards bone marrow-derived DCs in a transwell cell migration assay. The number of DCs that migrated toward neutrophil supernatant is represented. (D) DC migration was similarly assessed in response to neutrophil supernatants depleted of CCL3. *: p<0.05 as compared to values measured in response to supernatant of neutrophil incubated with medium alone. Data are expressed as mean number ± SEM of DC that migrated towards neutrophil supernatant (n = 3 per group). The results of a representative experiment out of three are shown.

Neutrophils have been reported to secrete chemokines in response to microbial stimuli [20]. First we determined whether *L. major* induced the transcription and secretion of DC-attracting chemokines in inflammatory neutrophils. *L. major*-recruited inflammatory neutrophils were purified by MACS and incubated with *L. major* and/or with IFNγ, an activator of neutrophils. Sixteen hours later, cells were collected for mRNA analysis, or twenty-four hours later, cell-free supernatant was analyzed for chemokines reported to attract immature DCs. Incubation of C57BL/6 neutrophils with *L. major* increased both CCL3 transcript levels and protein secretion, while only a very mild effect was seen in response to IFNγ alone (Figure 1A,B). In contrast, incubation of C57BL/6 neutrophils with *L. major* alone did not induce a significant increase of either CCL4 mRNA or protein, but incubation of neutrophils with IFNγ induced an increase in its mRNA and release (Figure 1A,B). IFNγ and to a lower extent *L. major*, independently induced CCL5 mRNA, with IFNγ alone inducing the highest release of CCL5. Interestingly, the presence of *L. major* significantly impaired the IFNγ-induced release of both CCL4 and CCL5 (Figure 1B).

To determine if *L. major* would induce similar transcription and secretion of DC-attracting chemokines from neutrophils in the *L. major*-susceptible BALB/c mice, *L. major* recruited inflammatory BALB/c neutrophils were treated and analyzed as described above. *L. major* did not induce significant level of chemokine transcription nor elevated release from BALB/c neutrophils (Figure 1A,B). Neither C57BL/6 nor BALB/c neutrophils secreted or transcribed CCL20 in response to *L. major*, IFNγ or both (data not shown).

The selective secretion of CCL3 by *L. major* peritoneally-induced inflammatory C57BL/6 but not BALB/c neutrophils (Figure 1) was also measured in inflammatory dermal neutrophils recruited in the ear dermis 24 hours after *L. major* inoculation (Figure S1).

Altogether, these results show that, once exposed to *L. major* promastigotes, C57BL/6 neutrophils secrete CCL3 a chemokine known to attract immature DCs, and that the production of chemokines by BALB/c neutrophils is significantly lower in response to the parasite.

We next tested the potential effect of neutrophil supernatant on chemoattraction of immature DCs using Transwell cell migration assays. Bone marrow-derived partially immature C57BL/6 and BALB/c DCs (MHCII^low^, CD40^−^, B7.1^−^, B7.2^low^) were deposited on the filter of a Transwell migration assay plate, the lower compartment containing the supernatants recovered from C57BL/6 or BALB/c neutrophils exposed or not to *L. major*. While robust chemo-attractive activity for BM-iDCs was detected in the supernatants of C57BL/6 neutrophils exposed to *L. major*, no similar strong chemo-attractive activity was detectable in the supernatants of BALB/c neutrophils exposed to *L. major* (Figure 1C).

To assess if this chemo-attractive activity was due to CCL3 two approaches were selected. First, CCL3 was depleted from supernatant of C57BL/6 mouse neutrophils exposed to *L. major*, and the CCL3-depleted supernatant monitored for its iDCs chemo-attractive activity, using the Transwell migration assay. Remarkably, a significant decrease of the BM-iDC migration towards the latter supernatant was measured (Figure 1D). Second, supernatants from CCL3^−/−^ C57BL/6 mouse neutrophils exposed to *L. major* were similarly tested and shown to display reduced chemo-attractive activity for +/+ BM-iDCs (data not shown).

In contrast, CCL3 depletion of BALB/c supernatants showed only a mild and not statistically significant effect on DC chemoattraction, in line with the low chemokine secretion and chemoattraction of *L. major*-stimulated BALB/c neutrophil supernatants (Figure 1D).

These results demonstrate that the CCL3 present in the supernatant of C57BL/6 neutrophils exposed to *L. major* promastigotes is the main chemokine attracting BM-iDCs *in vitro*.

### Recruitment of DCs in the ear dermis of C57BL/6 and BALB/c mice during the first 48 hours post *L. major* inoculation

A significant difference in chemokine secretion was measured in our ex vivo/in vitro analysis between *L. major*-stimulated C57BL/6 and BALB/c neutrophils, with consequence on the recruitment of iDCs. This prompted us to investigate whether similar differences were observed *in vivo*. To evaluate how neutrophil and chemokine release influence dendritic cell recruitment *in vivo*, the model of ear skin explants was used. *L. major* was delivered intradermally in the ear and at different time points post inoculation, ears were recovered, and further processed as ear explants. This ex vivo approach allows the evaluation of DC recruitment in the skin dermis [21],[22].

C57BL/6 and BALB/c mice were infected i.d. in the ear with *L. major*, and the number of cells that migrated out of infected ear skin explants was analyzed by FACS and quantified during the first 48 hours following parasite inoculation.

**Figure 2:**
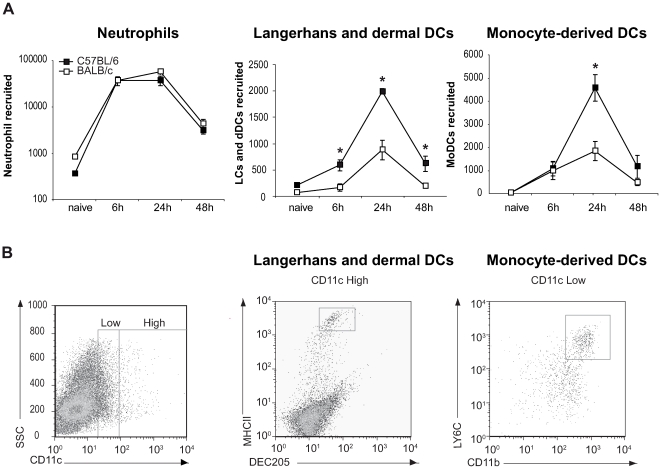
Higher number of DCs are recruited to the ear dermis of *L. major* infected C57BL/6 compared to BALB/c mice. (A) The number of neutrophils, Langerhans and dermal DCs, and monocyte-derived DCs emigrating from ear explants was measured at 0, 6, 24 and 48 hours post *L. major* inoculation *: p<0.05 comparing cell number in C57BL/6 versus BALB/c mice. Values from ear explants of six mice per group are expressed as mean values ± SEM . The data are representative of three separate experiments. (B) Gating strategy by flow cytometry of Langerhans and dermal DCs, and monocyte-derived DCs emigrating from *L. major*-infected C57BL/6 ear explants. For Langerhans and dermal DCs, MHCII and DEC205 positive cells were analyzed on CD11c^+^ gated cells. For monocyte-derived DCs, four colour FACS analysis was performed, CD11b^+^ and LY6C^+^ cells were analyzed on a CCD11c^dim^ and Ly6G^−^ gated cell population. A representative flow cytometry plot is shown.

A large number of neutrophils emigrated from the ear explant within hours of parasite inoculation and their number started to decrease 48 hours later (Figure 2A). As previously reported for *L. major* infected footpads [4],[5], at this early stage post parasite inoculation the neutrophil number did not differ between C57BL/6 and BALB/c mice. We then estimated the number of Langerhans cells (LC) and dermal DC (dDC), defined by FACS analysis by their high surface expression of CD11c, DEC205, and MHC Class II (Figure 2B). Already six hours after infection, the number of LC and dDC migrating out of the ear dermis was significantly higher in C57BL/6 than in BALB/c mice, with the highest difference occurring twenty-four hours post infection (Figure 2A). As monocyte-derived DC (MoDC) play an important role in *L. major* infection [23], this cell population, characterized as being Ly6G^−^, Ly6C^+^, CD11b^+^ and CD11c^dim^ (Figure 2B), was also analyzed by FACS. Twenty-four hours post *L. major* inoculation, a significantly higher number of MoDCs emigrated from C57BL/6 ear explants as compared to BALB/c ear explants (Figure 2A). Most of the DCs emigrating from the ears resulted from the *L. major* presence, while only a small percentage of DCs emigrated from the ears was due to needle-dependent injury, as illustrated by the low DC recruitment measured when medium alone was injected (Figure S2).

These results reveal a significant difference in DC migration during the first day post infection with *L. major* between C57BL/6 and BALB/c mice.

### Neutrophils are required for DC recruitment to the site of *L. major* inoculation in C57BL/6 mice

**Figure 3:**
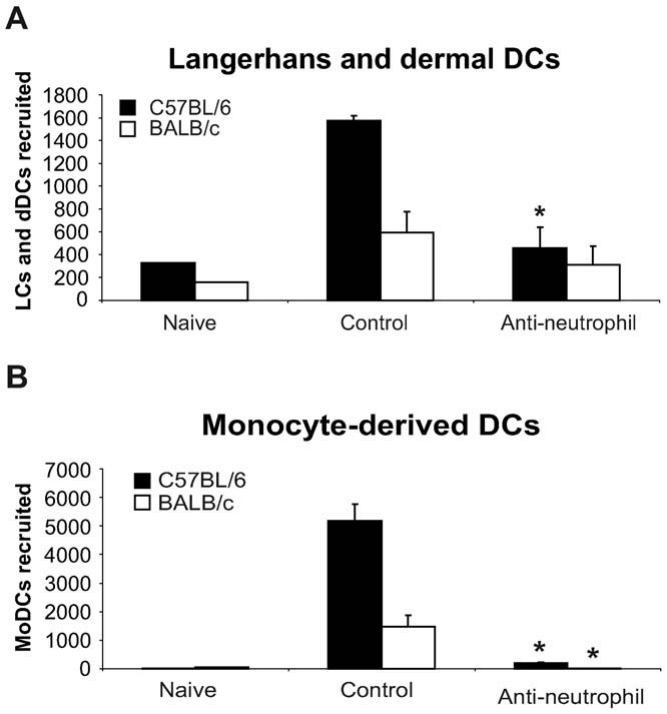
Neutrophils are essential for DC recruitment to the ear dermis following *L. major* promastigote inoculation. C57BL/6 and BALB/c mice were depleted of neutrophils by an injection i.p. of the NIMP-R14 anti-neutrophil mAb, or injected with a control mAb, 6 hours prior to infection i.d. with *L. major* stationary promastigotes. The number of (A) Langerhans and dermal DCs and (B) monocyte-derived DCs recruited in the ear dermis at 0h (naïve) or 24h following *L. major* inoculation was quantified and compared in mice depleted or not of neutrophils. Data are given as mean values ± SEM (n = 6 per group) and are representative of three experiments. * : p<0.05 between mice depleted or not of neutrophils.

In order to further investigate whether the difference in the number of DCs recruited during the first day post *L. major* inoculation in C57BL/6 and BALB/c mice correlated with their distinct neutrophil functional phenotype, mice of both strains were given i.p. a single injection of the neutrophil-depleting mAb NIMP-R14, and six hours later, *L. major* promastigotes were i.d. delivered in one of their ears. Twenty-four hours post *L. major* inoculation, the DC subsets sedimenting out of the ear explants were analyzed by FACS, quantified and compared to the ones sedimenting from ear explants prepared from mice that were given a control mAb. Depletion of neutrophil significantly decreased the number of LC and dDC and abolished the recruitment of MoDC in the ear skin dermis (Figure 3A,B). These results demonstrate an essential role for neutrophils in the early recruitment of DC following inoculation of *L. major*.

### CCL3 is the major neutrophil-derived DC-attracting chemokine at the site of *L. major* inoculation

Since neutrophils appeared to be essential for the recruitment of dendritic cells in the first days following infection with *L. major* and as we have shown *in vitro* that the CCL3 secreted by neutrophils was critical for the recruitment of DCs, we next sought to document whether this chemokine could play an essential role *in vivo*.

**Figure 4:**
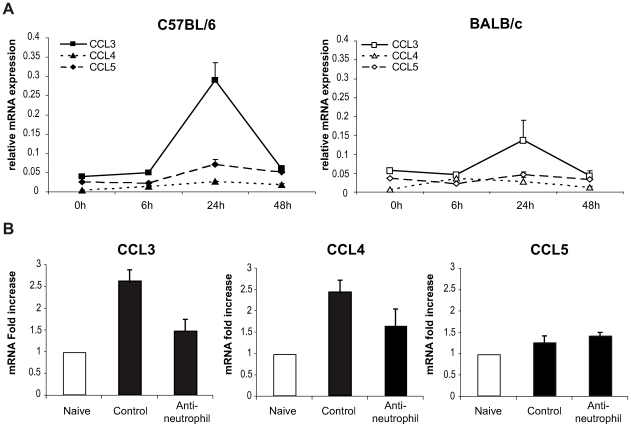
CCL3 is a major DC-attracting chemokine in the ear dermis one day post *L. major* inoculation. (A) C57BL/6 and BALB/c mice were infected with *L. major* in the ear dermis. mRNA expression of CCL3, CCL4 and CCL5 at the site of infection was measured 0, 6, 24 and 48 hours post infection by quantitative Real-time PCR and normalized relative to HPRT mRNA levels. Data are represented as the mean ± SEM mRNA transcript levels of individual infected ear (n = 6 per time point). One representative experiment of three is shown. (B) Twenty four hours post-infection, the ear chemokine mRNA level was compared in C57BL/6 mice that were given either the NIMP-R14 neutrophil depleting mAb or a control mAb 6 hours prior to *L. major* inoculation in the ear. Results are represented as fold increase in mRNA levels relative to levels measured in uninfected mice given a value of 1, and are representative of two experiments.

To this end, the level of chemokine mRNA was measured in *L. major* infected ears during the first 48 hours post infection. In C57BL/6 infected mice, CCL3 mRNA was strongly induced within 24 hours of *L. major* inoculation (Figure 4A), while significantly less CCL3 mRNA was induced in *L. major* infected BALB/c mice. *L. major* induced only a small increase in CCL4 and CCL5 mRNA at the site of infection (Figure 4A), while infection did not induce CCL20 mRNA (data not shown).

To investigate if neutrophils were responsible for CCL3 transcription at the site of infection in C57BL/6 mice, neutrophils were depleted with an injection of the NIMP-R14 mAb 6 hours prior to infection, and chemokine transcript abundance was measured in infected ears 24 hours post parasite inoculation. In *L. major* infected mice injected with a control mAb, CCL3 mRNA levels were increased significantly 24 hours after *L. major* inoculation while in neutrophil-depleted mice, CCL3 mRNA levels were much lower (Figure 4B). Among the three other chemokine transcripts - CCL4, CCL5, and CCL20 – monitored, only the CCL4 followed a transcript profile similar to the CCL3 one though with lower amplitude (Figure 4A). These data demonstrate that twenty-four hours post parasite inoculation in the ear, neutrophils contribute to most of the CCL3 and part of the CCL4 present during the first day of infection.

**Figure 5:**
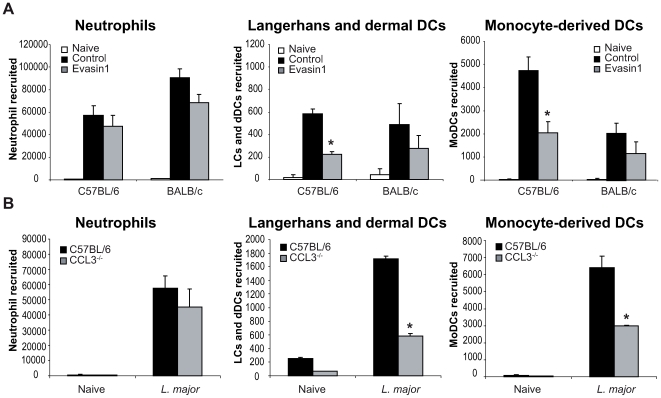
CCL3 is essential for early DC trafficking/recruitment to the site of infection following *L. major* inoculation. (A) C57BL/6 and BALB/c mice were injected i.p. with Evasin-1, a chemokine binding protein that neutralizes CCL3. Twenty four hours post *L. major* inoculation, the number of neutrophils, Langerhans and dermal DCs, and monocyte-derived DCs, spontaneously emigrating out of ear explants was measured and compared to that obtained from ear explants from mice similarly infected but which were not given Evasin-1. * : p<0.05 between mice treated or not with Evasin-1. (B) 24 hours after infection i.d. with *L. major*, the number of leukocytes emigrating out of ear skin explants of CCL3^−/−^ mice was compared to that measured in similarly infected C57BL/6 ear explants. The number of neutrophils, Langerhans and dermal DCs, and monocyte-derived DCs is presented as the mean ± SEM (n = 4 for Evasin-1 treated mice, and n = 6 for CCL3^−/−^ mice) and are representative of three experiments. *: p<0.05 between CCL3^−/−^ and C57BL/6 mice.

In order to directly establish that CCL3 acts as a key chemokine accounting for the recruitment of DCs in the *L. major*-loaded dermis, CCL3 was depleted in mice by treatment with Evasin-1 [24], a highly selective neutralizing chemokine binding protein with high affinity for CCL3 and to weaker affinity for CCL4 [25]. Mice were given Evasin-1 two hours prior to i.d. *L. major* inoculation in the ear, and DC mobilization in the infected ear explants was quantified as described above. Injection of Evasin-1 had no significant effect on the number of neutrophils that migrated out of ear skin dermis 24 hours after infection (Figure 5A). This allowed the investigation of the role of neutrophils on DC recruitment at the site of infection, under conditions when CCL3 secretion was neutralized. While injection of Evasin-1 into C57BL/6 mice resulted in significant decrease of emigration of both LC/dDC and MoDC from the ear explants, injection of Evasin-1 into BALB/c mice did not result in any similar phenotypic changes (Figure 5A).

To confirm the role of CCL3 in DC recruitment *in vivo*, C57BL/6 mice or mice genetically deficient in CCL3 (CCL3^−/−^) on the C57BL/6 genetic background, were infected with *L. major* i.d., and emigration from the ear explants assessed as described above. As measured in mice treated with Evasin-1, neutrophil recruitment was not significantly decreased one day post infection. However, recruitment of both LC/dDC, and MoDC was significantly reduced in CCL3^−/−^ mice (Figure 5B), confirming that CCL3 is a major chemokine involved in LC/dDC migratory properties as well as MoDC recruitment. These results strongly suggest that the CCL3 secreted by neutrophils contributes for a major part to early DC trafficking and/or recruitment in the dermis of *L. major* inoculated mice.

### Transfer of wild type neutrophils restores DC traffic/recruitment in CCL3^−/−^ C57BL/6 mice inoculated with *L. major*

**Figure 6:**
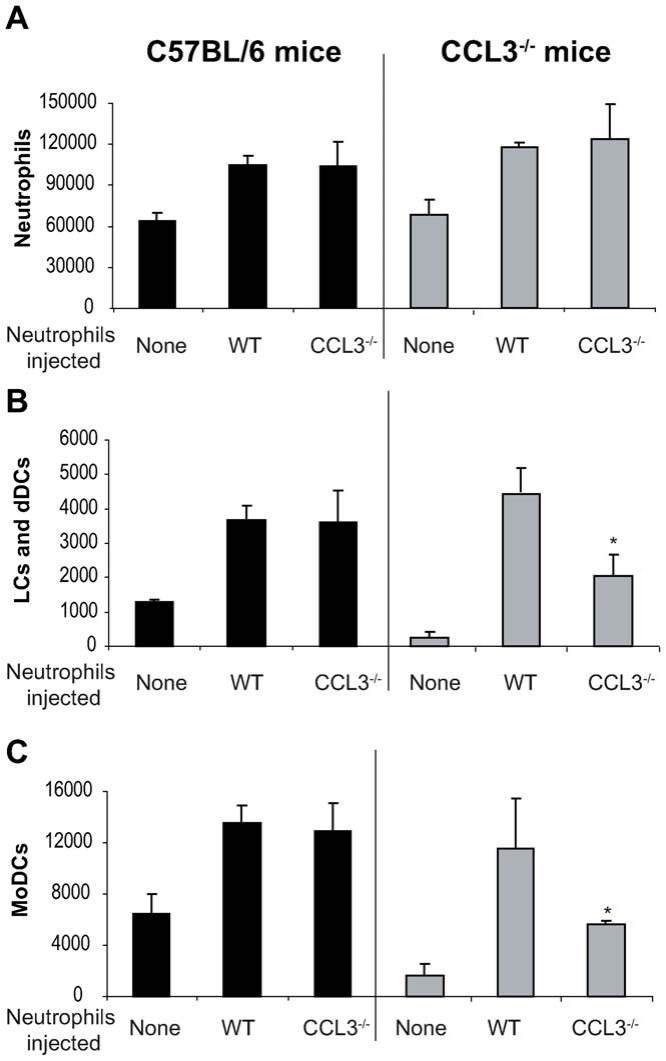
The CCL3 secreted by neutrophils is the major chemoattractant for DCs one day after *L. major* inoculation. Inflammatory neutrophils from C57BL/6 or CCL3^−/−^ mice were injected in the ear dermis of C57BL/6 or CCL3^−/−^ mice simultaneously with *L. major*. (A) The neutrophils, (B) Langerhans cells, dermal DCs, and (C) monocyte-derived DCs emigrating from the ear explants were monitored 24 hours post inoculation. Data obtained from four individual mice are expressed as mean values ±SEM. The results are representative of three experiments. * : p<0.05 compared with injection with WT neutrophils.

So far, our data suggest that neutrophils and CCL3 contribute significantly to the early DC trafficking/recruitment to the site of *L. major* inoculation in C57BL/6 mice. In order to monitor whether neutrophils were indeed the major source of CCL3 that mediates DC recruitment, we transferred either C57BL/6 wild type (WT), or CCL3^−/−^ neutrophils into CCL3^−/−^ mice that were given i.d. *L. major*, and twenty-four hours later, leucocytes emigrating from the ear explants were analyzed by FACS. The number of neutrophils did not differ significantly after injection of WT or CCL3^−/−^ neutrophils into C57BL/6 and CCL3^−/−^ mice (Figure 6A). However, injection of WT neutrophils into ears of CCL3^−/−^ mice strongly increased the number of LC/dDC and MoDC migrating out of the the ear explants, to levels that were comparable to those measured in WT mice injected with WT or CCL3^−/−^ neutrophils (Figure 6B,C right panels). Even though injection of CCL3^−/−^ neutrophils into CCL3^−/−^ mice also increased the number of LC/dDC and MoDCs, the levels attained were significantly lower than those obtained when CCL3^−/−^ mice were injected with WT neutrophils. These results demonstrate that the CCL3 secreted by neutrophils plays an important role in the early trafficking/recruitment of DCs cells to the site of infection during the first days of *L. major* infection.

### Depletion of CCL3 during the first days post *L. major* injection delays the onset of *L. major*-specific Th1 immune response

**Figure 7:**
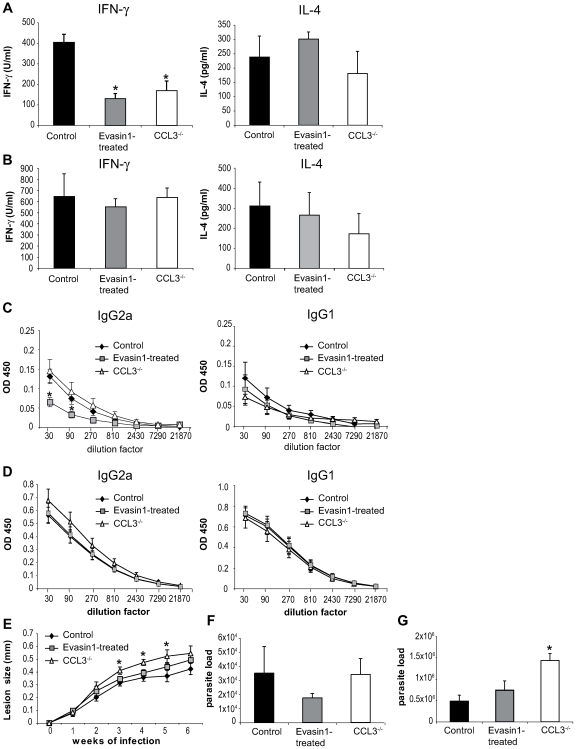
Depletion of CCL3 during the first day post *L. major* inoculation delays the development of the *L. major*-induced Th1 immune response. CCL3^−/−^ mice, and C57BL/6 mice in which CCL3 was blocked by injection of Evasin-1 for the first five days post *L. major* inoculation, were inoculated s.c. with 3×10^6^ *L. major*. CD4^+^ draining lymph nodes cells were prepared at days 15 (A) and 40 (B) post *L. major* inoculation and co-incubated with UV irradiated *L. major*. The resulting supernatants were monitored for their IFNγ and IL-4 content by ELISA. Data are the mean of triplicate measurement ± SEM of cytokines (n = 8 mice per group). Fifteen (C) or forty (D) days post-infection *L. major*-specific IgG2a and IgG1 Ab production was quantified in sera from infected mice. Data are the mean triplicate OD ± SEM of serum dilution. (E) Course of infection in CCL3^−/−^ and Evasin-1 treated mice. Evolution of lesion size was monitored every week with a Vernier caliper. Each point represents the mean lesion size ±SEM (n = 8 per group). Parasite burden was measured by limiting dilution assay (LDA) fifteen (F) and forty (G) days post inoculation (n = 8 per group). The experiments are representative of five (15 days) and three (6 weeks) independent experiments.

To investigate the role of CCL3 in the onset of the immune response in C57BL/6 mice, CCL3 was inhibited during the first five days post *L. major* inoculation by daily administration of Evasin-1. Fifteen and forty days post infection, the development of T helper immune response was assessed. *L. major*-specific IFNγ and IL-4 cytokine production was measured in draining lymph node CD4^+^ T cells, and immunoglobulin isotype switching was measured in the serum. Fifteen days post infection the transient depletion of CCL3 resulted in almost total abolition of IFNγ secretion, but no significant difference in IL-4 levels (Figure 7A). The low IFNγ levels correlated with a significant decrease of *L. major*-specific IgG2a and a milder but not statistically significant increase in IgG1 serum levels in Evasin-1 treated mice (Figure 7C). Low IFNγ secretion was also measured fifteen days post *L. major* inoculation in CCL3^−/−^ mice (Figure 7A and 7C), in line with results from a previously published study [26], but IgG2a levels did not differ significantly from C57BL/6 controls, while IgG1 levels were slightly increased (Figure 7C). Of note, very low levels (<100pg/ml) of IL-13 and IL-17 were measured in supernatant of *L. major*-restimulated CD4^+^ T cells, with no difference between the groups (data not shown).

Six weeks post *L. major* inoculation, high levels of IFNγ with correspondingly high levels of *L. major*-specific IgG2a, and low levels of IL-4 and IgG1 characteristic of a Th1 immune response were measured, with no statistically significant difference being noted between the groups (Figure 7B, 7D). Mice treated with Evasin-1 developed slightly larger lesion than control mice but smaller lesions than CCL3^−/−^ mice, with no statistically significant difference (Figure 7E). In contrast, development of lesion was markedly increased in CCL3^−/−^ mice compared to C57BL/6 controls, and CCL3^−/−^ mice harboured higher parasite load within their lesions, with a significant difference compared to Evasin-1 treated or control mice (Figure 7G).

## Discussion

In this study, we focused on the role of neutrophils in the recruitment of DCs to the site of *L. major* inoculation, one main question addressed being whether neutrophils could transiently and locally contribute to the Langherans cell/dermal dendritic cell trafficking as well as to the recruitment of monocytes, the latter being capable to be programmed to either macrophages or monocyte-derived DCs. Our results demonstrate a previously unappreciated role of primary neutrophil extravasation, and of the CCL3 released by neutrophils in the rapid recruitment and trafficking of LC/dDC as well as of monocyte-derived DC to the site of *L. major* inoculation. We report here for the first time that the selective secretion of CCL3 by neutrophils is critical *in vivo* for the recruitment of DCs to the site of *Leishmania* inoculation, as revealed by markedly reduced recruitment of DCs in mice with pharmacological neutralization or absence of CCL3, and in neutrophil-depleted mice. This decrease was restored in CCL3^−/−^ mice by the co-injection of WT C57BL/6 neutrophils together with the parasite.

CCL3 has been reported to attract neutrophils, however, our data clearly show that this chemokine does not contribute significantly to the first wave of neutrophil migration following *L. major* inoculation, as the number of neutrophils recruited to the site of parasite delivery one day post infection was not significantly affected by either the absence or the neutralization of CCL3.

Previous studies performed *in vitro* reported the transcription or/and secretion of DC attracting chemokines by neutrophils. Human neutrophils were shown to secrete molecules involved in attraction of immature DCs such as defensins [27], and CCL3 following LPS stimulation *in vitro* [28]. Murine neutrophils were also reported to transcribe CCL3, CCL4, CCL5 and CCL20 mRNA in response to *Toxoplasma gondii* exposure *in vitro*, with the highest induction of CCL5 mRNA [29]. In the present study, *L. major* induced the highest transcription and secretion of CCL3, low level of CCL5, and no transcription nor secretion of CCL4 and CCL20 *in vitro*. Thus, distinct pathogens can elicit different chemokine transcription patterns in neutrophils, and as reported here, the same pathogen can induce distinct chemokine release depending on the genetic background of the host. In this line, induction of TLRs in response to *L. major* was previously reported to differ in neutrophils from C57BL/6 and BALB/c mice, respectively resistant or susceptible to *L. major* [9].

The coordinate expression of chemokines and their receptors has been shown to be important in protective immunity to infection with *L. major*. While most studies have focused on chemokine expression in draining lymph nodes, only a few have investigated chemokine expression at the site of parasite inoculation: CCL2 and CCL3 mRNA expression were reported to be elevated already one day post infection in *L. major* infected footpads of C57BL/6 mice [30]. CCR2, the receptor for CCL2 and other monocyte chemoattractant proteins, was thought to be required for the generation of a protective immune response against *L. major*, but the differential outcome observed in CCL2^−/−^ and CCR2^−/−^ mice following *L. major* infection suggests that ligands other than CCL2 were involved in this protection [26],[31]. We also observe that in the presence of *L. major*, low level of CCL5 is secreted by neutrophils, but we show that it is not the major neutrophil-secreted chemokine involved in the recruitment of DCs. Indeed, during *L. major* infection, CCL5 expression was reported to increase selectively in C57BL/6 compared to BALB/c mice, but mainly in the late phase of infection [32]. We also report here a small transient increase in CCL4 and CCL5 mRNA one day post *L. major* inoculation at the site of infection in C57BL/6 mice, but CCL3 clearly showed a much higher level of transcriptional induction in these mice. Altogether, these and our studies reveal the importance of the tight control and timing of chemokine secretion during the first days post *L. major* inoculation.

How can the different levels of CCL3 released by C57BL/6 and BALB/c neutrophils exposed to *L. major*, affect the subsequent development of the immune response? Our data demonstrate that the early secretion of CCL3 has an impact on the development of the adaptive immune response, first through the recruitment of DC, and second, possibly through their activation. Whether and how neutrophils impact on the subsequent DC functions that account for T lymphocyte signaling is currently under investigation. Neutrophils have indeed been reported to deliver maturation and activation signals to DCs in BCG and *Toxoplasma gondii* infection [29],[33], and neutrophils were shown to associate with immature DC through interactions between DC-SIGN on immature DC and specific glycans on neutrophils [34]. In addition, neutrophil-derived ectosomes have been reported to interfere with the maturation of MoDCs [35]. Whether these DC subsets reach the skin-draining lymph node remains to be established, but it is plausible that they do, providing signals to T lymphocytes. In this line, following *L. major* inoculation, the early absence of CCL3 had consequences on the Th1 immune response normally developing fifteen days post parasite inoculation in the draining lymph nodes of C57BL/6 mice, preventing most of the IFNγ secretion by draining lymph node CD4^+^ cells. Further investigations will allow deciphering the mechanism involved in the early though transient inhibition of Th1 response following early neutralization of CCL3, and in the stepwise neutrophil-dependent processes that allow DCs to traffic from the dermis to the skin- draining lymph node at the very early stage post *L. major* promastigote delivery.

BALB/c and C57BL/6 neutrophils exposed to *L. major* have been reported to differ in the induction of cytokine secretion, with C57BL/6 neutrophils secreting IL-12, and BALB/c neutrophils secreting IL-12 p40 homodimers, blocking IL-12 signalling [9]. We report here the selective secretion of CCL3, a chemokine reported to induce IL-12 secretion in macrophages, by C57BL/6 but not BALB/c neutrophils. These differences in cytokine and chemokine secretion in *L. major*-exposed C57BL/6 and BALB/c neutrophils explain, at least in part, their distinct contribution to the subsequent development of T helper cells in the draining lymph node.

Several DC subsets have been reported to be involved in the development of the *L. major* protective immune response [10],[11] (reviewed in [12]), and infection-induced inflammatory reactions include a sharp increase in DCs at the site of parasite inoculation. *L. major* has been shown to be phagocytosed by dermal DCs [36], and *Leishmania* antigens have been reported to be transported by dermal DCs rather than by LCs [37]. Recently, de novo differentiation of monocytes into DCs, and the crucial importance of these migratory dermal monocyte-derived DCs in controlling the development of a protective CD4^+^ Th1 type of immune response has been demonstrated in *L. major* infection [23], even if a role for resident lymph node DCs is not excluded [38]. Thus the role of neutrophil-derived chemokines, together with the important contribution of CCL3 in the early recruitment of MoDC at the site of infection reported in the present study, emphasize the importance of neutrophils in recruiting the cells contributing to the priming of CD4^+^ Th1 cells that are essential in efficient protection against *L. major* infection.

Natural transmission of *L. major* is occurring during the bite of an infected sandfly. When mouse ears are exposed to *Leishmania*-hosting sand flies, neutrophils are rapidly recruited to the site of parasite inoculation, an early phenotypic trait also observed after intradermal needle inoculation of *L. major* [6]. It will be important to compare the two experimental systems and to explore whether factors derived from the sandfly may contribute to the early and transient wave of leucocytes as well as to their short term functions.

In conclusion, neutrophil and neutrophil-produced CCL3 appear crucial in the early recruitment of dendritic cells in the dermis, that will further direct the development of an adaptive immune response to *L. major*. Therefore, strategies interfering with these factors could represent a novel way to shape immune responses to pathogens.

## Materials and Methods

### Mice and parasites

Female BALB/c and C57BL/6 mice were purchased from Harlan Olac Ltd. (Bicester, UK). CCL3^−/−^ mice were purchased from Jackson laboratory (Bar Harbor,USA). All mice were bred in the pathogen-free facility at the BIL Epalinges Center and used at 6 week of age. *L. major* (LV 39 MRHO/Sv/59/P strain) were maintained and grown as previously described [2]. All animal experimental protocols were approved by the veterinary office regulations of the State of Vaud, Switzerland, authorization 1266.3 to FTC, and experiments were performed adhering to protocols created by this office.

### Isolation of neutrophils

Mouse inflammatory neutrophils collected by peritoneal lavage 4 hours post infection i.p. of 5.10^7^ stationary phase *L. major*, were isolated and purified using MACS-positive selection as previously described [9]. Purity of neutrophils was >98% as assessed by FACS and Diff-Quick (Dade Behring) staining of cytospins. Each experiment was validated using FACS sorted neutrophils positively gated through 1A8 labeling and negatively gated with a cocktail of mAbs (against CD3, CD49b, B220, F4/80, and CD11c, see below).

### Culture of neutrophils and chemokine detection

Neutrophils were cultured in RPMI-1640 media supplemented with 10% FCS and antibiotics (2.5×10^6^ cells/ml) in the presence or absence of *L. major* metacyclic promastigotes (at a 5∶1 parasite∶cell ratio). The protease inhibitor aprotinin (0.4 µg/ml, Sigma Chemical Co., St. Louis, MO, USA) was added to the culture to facilitate cytokine detection. Chemokine concentration in the culture supernatant was quantified by ELISA using kits from R&D systems.

### mRNA isolation, cDNA synthesis, and quantitative RT-PCR

Inflammatory neutrophils cultured *in vitro* under different conditions were harvested, mRNA extracted, cDNA synthesized and quantitative RT-PCR performed as previously described with a LightCycler system (Roche) [9]. Each cytokine transcript was normalized to the value of the hypoxanthine phosphoribosyltransferase endogenous control, represented as arbitrary values. The primers for the real time PCR were the following: **CCL3**: F 5′ CCA AGT CTT CTC AGC GCC AT 3′, R 5′ TCC GGC TGT AGG AGA AGC AG 3′, **CCL4**: F 5′ TCT TGC TCG TGG CTG CCT 3′, R 5′ GGG AGG GTC AGA GCC CA 3′, **CCL5** [39], **CCL20** F5′ CTT GCT TTG GCA TGG GTA CT 3′, R 5′ GTC TGT ATG TAC GAG AGG CA 3′.

### CCL3 depletion

CCL3 depletion in neutrophil supernatants. Neutrophil supernatant were placed on a plate coated with antibody from the CCL3 ELISA kit (R&D systems). Depletion in supernatant was checked by ELISA. For *in vivo* depletion, mice were treated with Evasin-1, a CCL3 blocking protein, engineered by Merck-Serono [24]. 10 µg of Evasin-1 were injected intraperitoneally 2h before injection of the parasite. As controls, mice were injected with a similar regimen of PBS.

### Generation of bone marrow derived immature DCs

Bone-marrow cells were cultured in RPMI-1640 media supplemented with 10% FCS, antibiotics and 30% GM-CSF for 6 days, as previously described [40]. At day 6, the maturation of DCs was checked by FACS, measuring the levels of MHC ClassII, CD40, B7-1 and B7-2 surface molecules.

### Transwell cell migration assay

Supernatant from neutrophils cultured under different conditions were placed in the lower compartment of a transwell plate (96 well plate, 3.2mm diameter 5um pore size, ChemoTx System, NeuroProbe, UK). 10^5^ DCs were put on top of the filter. After 2h of incubation at 37°C, the number of DCs that migrate towards the supernatant were counted on Neubauer chambers using trypan blue.

### Depletion of neutrophils

Mice were given i.p. -6h before *L. major* inoculation 250 µg of the NIMP-R14 mAb, a rat IgG2b mAb that selectively binds to mouse neutrophils [41]. This treatment was previously reported to deplete selectively neutrophils for three days [8]. As controls, mice were given i.p. the RR3-16 mAb against the Vα3.2 chain of the T-cell receptor (RR3-16, gift of R. MacDonald, Ludwig Institute for Cancer Research, Epalinges, Switzerland).

### *L. major* intradermal inoculation in the mouse ears and leucocyte emigration from ear skin explants

Mice were given intradermally into ear PBS or 10^6^ stationary phase *L. major* promastigotes. Six, 24, 48h post *L. major* inoculation mice were sacrificed, the ventral and dorsal sheets of the ear were separated with forceps, the two leaflets being transferred dermal side down in a plate containing RPMI-1640 media supplemented with 10% FCS and antibiotics at 37°C. The leukocyte populations emigrating spontaneously over 14 hours from the ear explants were then counted and stained for a FACS analysis [22]. In selected experiments, dorsal and ventral sheets of the ears were separated and the dermal side was digested with 0.1 mg/ml of Liberase TL (Roche) for 2h at 37°C. Ears from 7 mice were pooled, cut into pieces and filtered through a 40 µm filter, washed and processed for FACS sorting. Sorted neutrophils were further incubated in RPMI medium for 24 hours. Chemokine presence in neutrophil-free supernatant was then analyzed by ELISA.

### FACS analysis of the leukocyte populations emigrating from the ear explants

Leukocytes emigrating from the ear explants were processed for cell surface staining. The mAb 24G2 was used to block FcRs. For analysis of cell populations several mAbs were used: PE conjugated anti-LY6G (clone 1A8), anti-CD3 (clone G4.18), anti-CD49b (clone DX5), anti-MHCII (clone M5/114.15.2), Cyc conjugated-strepatvidin, PE conjugated, APC and Cyc conjugated anti-CD11c (clone N418), FITC conjugated anti-LY6-C (clone AL-21), Cyc conjugated anti-CD11b (clone M1/70), all mAbs from e-bioscience, SanDiego, CA, US; anti-DEC205 (clone NLDC-145, AbB Serotec,UK, Ldt). Biotinilated anti-F4/80 (clone C1∶A3-1,CEDRALANE, Canada). Cells were analyzed with a FACScan (3 colors) or FACSCalibur (4 colors) (BD Biosciences, Mountain View, CA, USA) and analyzed with the program FlowJo (Tree Star. Inc., Ashland, OR, USA).

### Intradermal co-inoculation of neutrophils with *L. major* promastigotes

Four hours post i.p. inoculation of *L. major* peritoneal lavage neutrophils were purified by MACS. 10^6^ C57BL/6 or CCL3^−/−^ neutrophils were co-injected in the ear dermis with 10^6^ stationary phase *L. major* promastigotes. Twenty-four hours later, mice were sacrified, ears were processed as above and emigrating cell populations analyzed by FACS.

### Detection of immune response in CCL3^−/−^ mice and in mice transiently depleted of CCL3

3×10^6^ *L. major* were inoculated in the footpad of either CCL3^−/−^ mice or +/+ mice in which CCL3 was blocked during the first five days of infection by daily injection of Evasin-1. Mice were sacrificed 15 and 40 days post- *L. major* inoculation in the footpad. Draining lymph node CD4^+^ T cells were isolated by MACS (Miltenyi Biotec), and cultured in the presence of irradiated C57BL/6 splenocytes ± UV-irradiated *L. major* promastigotes. Cytokine levels were measured by ELISA. Sera obtained at different time points post *L. major* inoculation were tested for *L. major*-binding IgG1 and IgG2a, and parasite burden was determined by limiting dilution assay as previously described [42].

### Statistical analysis

Data were analysed using the Student's t-test for unpaired data.

## Supporting Information

Figure S1 Secretion of chemokines by neutrophils of the ear dermis 24 hours post *L. major* promastigote inoculation. *L. major* stationary phase promastigotes were inoculated i.d. in the ears of C57BL/6 or BALB/c mice (n = 7/strain). 24 hours later, ears were digested, inflammatory neutrophils labeled and FACS sorted, as described in the Materials and Methods section. Neutrophils were incubated for 24 hours in medium, and chemokine secretion was measured in culture supernatant by ELISA. * p<0.05 between BALB/c and C57BL/6 neutrophil supernatant.
(0.53 MB EPS)

Figure S2 DC recruitment resulting from *L. major* versus needle injury. The number of neutrophils, Langerhans/dermal DCs, and monocyte-derived DCs emigrating from ear explants was measured at 0, 6, 24 and 48 hours post *L. major* inoculation (solid lines), or post injection of a similar volume of DMEM medium (dotted lines) *: p<0.05 comparing cell number in C57BL/6 versus BALB/c mice. Values from ear explants are expressed as mean values ± SEM (n = 8).
(0.73 MB EPS)
